# A new health economic measure for improving the health and wellbeing of older Australians in subacute care settings: a study protocol

**DOI:** 10.1186/s12877-026-07558-9

**Published:** 2026-04-27

**Authors:** Jenny Cleland, Kiri Lay, Jyoti Khadka, Zoe Adey-Wakeling, Craig Whitehead, Dina LoGiudice, Julie Ratcliffe

**Affiliations:** 1 Health and Social Care Economics, Caring Futures Institute, College of Nursing and Health Sciences, Flinders University, Adelaide, South Australia Australia; 2 Department of Rehabilitation, Flinders Medical Centre, Southern Adelaide Local Health Network, Adelaide, South Australia Australia; 3 College of Medicine and Public Health, Flinders University, Adelaide, South Australia Australia; 4 Division of Rehabilitation, Aged Care and Palliative Care, Flinders Medical Centre, Noarlunga Hospital and REPAT Precinct Southern Adelaide Local Health Network, Adelaide, South Australia Australia; 5 Department of Medicine, Royal Melbourne Hospital, University of Melbourne, Melbourne, Victoria Australia

**Keywords:** Older adults, Quality of life, Subacute care, Preference-based measures, Economic evaluation

## Abstract

**Background:**

The measurement and valuation of quality of life for older people is critical for economic evaluation and for person-centred quality assessment across health and aged care sectors. However, most existing quality of life (QoL) measures were developed for younger populations and do not encapsulate what is important to older people’s QoL. The Quality of Life – Aged Care Consumers (QOL-ACC), a preference-based quality of life measure, co-designed with older Australians in aged care, addressed this gap and is now part of the National Aged Care Quality Indicator Program. Health and aged care systems for older people overlap as many individuals require a continuum of care that spans both sectors. In Australia, the health care system is predominantly occupied by older people, with subacute services the fastest growing area. Subacute care is a specialised multi-disciplinary care that supports recovery and functional optimisation where the main goal is maximisation of QoL. Building on our previous work in aged care, this study will develop and validate a QoL measure tailored to older people receiving subacute services, supporting improved care and informed policy making decisions.

**Methods:**

This study consists of three stages. Qualitative interviews with older people in subacute care settings to assess the face validity and content validity of the QOL-ACC measure using a think-aloud approach. These findings will inform revisions to the QOL ACC, which will then be tested using an iterative interviewing process. The revised QOL-ACC measure will then be validated for older people in subacute settings through rigorous psychometric assessments. Finally, a preference-based scoring algorithm to enable the calculation of quality-adjusted life years (QALYs) will be developed.

**Discussion:**

Quality improvements are needed across the health care system, particularly for older people, who may have complex health needs. However, their voices are often not heard, as no suitable preference-based QoL measure exists to monitor their QoL. This study will co-develop and validate the first preference-based QoL measure for older people in subacute settings, enabling the application of the QOL-ACC across both health and aged care systems leading to better informed decision-making and maximising QoL outcomes for older people.

## Background

Population ageing is placing increasing pressures on the health care system, resulting in reduced quality care and negatively impacting the health and wellbeing of older people [1]. In Australia, older people (65 years and over) are the fastest growing age cohort with 1 in 6 Australians aged 65 and over, and the proportion of older people aged 85 years and over living with physical frailty and dementia is rapidly rising [2]. The Australian health care system is predominantly occupied by older people, with people aged 65 years and over (17% of the total population) accounting for 44% of total hospitalisations and 52% of patient days [3]. Subacute care is a specialised multidisciplinary treatment for individuals who need skilled intensive short-term care to regain functional independence, and includes services such as rehabilitation, palliative acre, geriatric evaluation and management and psychogeriatric care [4]. Subacute services are mainly accessed by older people and represent the fastest growing area of the health system, with rehabilitation services the most accessed subacute care service by older people [3].

Health and aged care systems are closely interconnected, often forming a continuous pathway of care for older adults [5]. This connection is especially evident in subacute care, where optimising QoL is the key focus [6]. Subacute care supports recovery and functional optimisation in older people and acts as a critical bridge between hospital care and residential aged care [2]. Residential aged care settings provide multidisciplinary support, including rehabilitation for individuals who do not require inpatient care. These systems also intersect during key transition points, such as when individuals move from hospital to home or into residential care [5].

Person-centred care is widely recognised as the foundation of high-quality health care. Person-centred care involves placing the older person and their preferences at the centre, enabling services to provide not only clinical care but care that meets the individual’s physical, social and psychological needs, maximising the older person’s QoL [7]. However, there is an urgent need for quality improvements throughout the health care system, particularly for older individuals whose complex health conditions are often overlooked and whose voices are rarely heard [8]. Despite their substantial use of subacute services, the system lacks tailored, routine measures for monitoring and reporting the QoL of older people.

Preference-based QoL measures can be applied in quality assessment and are also suitable for application in economic evaluation as they consist of a preference-based scoring algorithm that can generate quality adjusted life years (QALYs) [9]. QALYs can be used to quantify the effectiveness of an intervention by allowing for both QoL and quantity of life to be measured simultaneously within a single outcome [9]. However, most QoL measures applied in health economic research with older people are typically developed with and for younger populations, with a narrow focus on health status alone [10, 11], meaning they do not encapsulate the broader aspects of QoL aspects important to older people [12]. Recently, a new preference-based QoL measure was developed from the ground up with older Australians receiving aged care services – the Quality of Life-Aged Care Consumers (QOL-ACC) [13–15]. The QOL-ACC is the first co-designed measure with older people accessing aged care services globally and bridges an important gap by including both health-related and psychosocial aspects of QoL in a single measure. The QOL-ACC consists of six dimensions (mobility, pain management, emotional wellbeing, social connections, independence, activities) rated on a 5-point frequency scale (“all of the time” to “none of the time”) [15].

The QOL-ACC is validated for use in aged care settings [16, 17] and has recently been incorporated nationally in Australia by the Federal Department of Health, Disability and Ageing through the Mandatory Aged Care Quality Indicator Program [18]. Consequently, for the first time in the history of Australia’s aged care system, the voices of > 240,000 residents from > 2,700 facilities are now being used for informing quality assessment processes and quality improvement in aged care [18]. This landmark reform means there is an opportunity to develop, validate and integrate similar measures for older people in health care settings, in particular subacute care settings such as rehabilitation, geriatric evaluation and management services, and transition care where the maximisation of older people’s QoL is a key outcome [6].

The overarching aim of the study is to further develop, validate and implement the QOL-ACC for the health system. This research study will build upon the existing QOL-ACC measure for aged care by working with older people in subacute care settings to adapt, validate and implement the QOL-ACC for the routine assessment and monitoring of QoL in this setting. It is intended that the findings from this study will assist policy makers and practitioners across the health system in implementing person centred care and improving the quality of care experience and the QoL of older people.

## Methods/design

This study forms part of a larger study that has been externally peer-reviewed and funded by a NHMRC Investigator Grant (2024/GNT2033277).

**Fig. 1:**
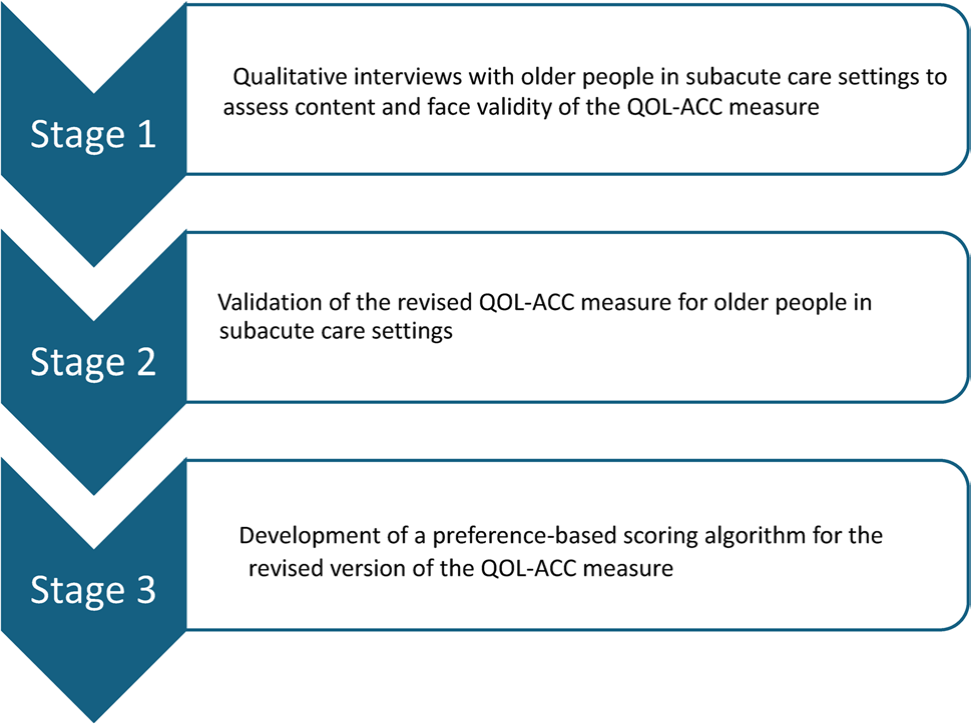
Overview of study

This study consists of three distinct stages (Fig. 1):

1. A series of in-depth face to face qualitative interviews with older people in subacute care settings to assess content and face validity of the QOL-ACC measure.
2. Psychometric assessment and validation of the revised QOL-ACC measure for application with older people in subacute care settings.
3. Development of a preference-based scoring algorithm for the revised version of the QOL-ACC measure.

### Stage 1 – qualitative interviews to assess content and face validity of the QOL-ACC measure

#### Participants and setting

Interviews (*N* = 20) will be conducted with older people in inpatient subacute care settings in South Australia. Participants will be aged 65 years and over, a current recipient of an inpatient subacute service and be able to read, speak and understand English. Participants will be purposively sampled to maximise inclusivity to reflect a range of socio-demographic characteristics ensuring rich in-depth data is pertinent.

#### Recruitment

Upon admission to the unit, a health care professional will provide a participant information sheet about the study to eligible participants. Interested participants will provide verbal consent to the health care professional for the researcher to approach them and provide more information. Interested participants will be given time to discuss their participation with family and friends before providing formal consent to the researcher. The interview will take place at a mutually agreed time in the participants room or in the participant’s preferred location within the unit.

#### Procedure

Interviews will be semi-structured, and the interviewer will follow an interview schedule. The interview will be conducted face-to-face in the unit. All interviews will be audio-recorded and transcribed verbatim by a professional transcriber. The interview will consist of 3 sections: socio-demographic questions, completion of the QOL-ACC and follow up questions.

The interviews will adopt a think-aloud approach that will involve the participants speaking out loud to articulate their thoughts whilst completing the paper-based version of the QOL-ACC. Both concurrent (asking participants to think aloud whilst completing the survey) and retrospective (if the participant did not verbalise their thoughts asking them after completion to revisit their thoughts in relation to their responses) techniques will be adopted. The think-aloud approach has successfully been used in previous research with older people where the aim of the research is to investigate older people’s understanding of QoL surveys [19–21]. The follow-up questions will explore participant’s comprehensibility, item wording issues, missing items, recall period issues, and suggestions for improvements to the measure.

#### Analysis

Data will be analysed using NVivo [22] and coded based on Tourangeau’s cognitive theory of survey response, comprising four key decision criteria – question comprehension, retrieval, judgement and response selection [23]. An inductive thematic approach will be conducted within the framework to identify common themes and patterns and for any other data arising from the interviews.

This analysis will inform whether any revisions to the existing QOL-ACC measure are required to ensure its applicability for subacute care settings. If revisions are required, testing of the revised measure will be conducted using an iterative interviewing approach with a new sample of older consenting participants currently receiving subacute care services. It is anticipated that there will be 3–4 rounds of interviews, with approximately five participants per round. Participants will be presented with a paper-based revised version of the QOL-ACC with the proposed modifications. The interview protocol will be semi-structured and will include a series of questions to assess relevance and comprehensibility. After each round of interviews, findings will be analysed for ongoing response issues based on the Tourangeau framework [23], and content validity criteria such as comprehensibility, clarity, interpretation and sensitivity (appropriateness of wording for the target population). The revised items will then be taken forward into the next round. This process will continue until the revised measure is finalised, whereby participants understand and interpret items as intended and the revised descriptive system is considered fully relevant to the older population in subacute care settings. Recruitment for these interviews will align with the process of recruitment for the first stage of qualitative interviews.

### Stage 2 – validation of the revised version of the QOL-ACC measure

An important stage in the further development and application of the QOL-ACC measure for the health system involves testing the psychometric properties, including validity and reliability. This approach will follow international best practice guidance [24, 25], and the rigorous psychometric methods previously successfully applied in the validation of the QOL-ACC measure [15–17].

#### Participants and setting

Older adults who are engaged with subacute care services (*N* = 200) will be recruited from sites across South Australia and Victoria. Participants will be aged 65 years and over and be able to read and understand English. Participants will be purposively sampled to maximise inclusivity to reflect a range of socio-demographic characteristics in subacute settings.

#### Recruitment

The recruitment process will be the same as the process outlined in stage one.

#### Procedure

Participants will be given a survey to complete comprising of socio-demographic questions, the revised version of the QOL-ACC, and two further QoL measures; the Adult Social Care Outcomes Toolkit (ASCOT) [26] and the EuroQol 5-Dimension 5-Level (EQ-5D-5 L) [27].

#### Analysis

All data will be analysed using STATA Version 19 [28]. Feasibility will be assessed by examining the proportions of missing data and floor/ceiling effects. Construct validity, the extent to which scores from measures represent the underlying construct they propose to measure [29]. This will involve assessing the convergent and known-group validity of the revised QOL-ACC measure. Convergent validity will examine whether the QOL-ACC correlates with the other two measures (ASCOT [26] and EQ-5D-5 L [27]) that claim to be measuring a related concept, and if so to what extent. Assessing convergent validity is achieved by making prior hypotheses of the expected correlations between the measures and their items and then testing the hypotheses [29–31]. Known group validity assesses the extent in which a measure can discriminate between groups that have known different characteristics and expected to have different QoL scores. This will be examined by identifying the extent of the differentiation between older people receiving subacute care services with different levels of self-reported health and between older people receiving subacute care services with different levels of self-reported QoL measured using the global QOL one-item measure and the global self-rated health one-item measure [32, 33]. Reliability will be assessed through Cronbach’s alpha and item scale correlations to determine internal consistency. Further, test-retest reliability will be assessed in a sub-group (*N* = 50) of participants will complete the QOL-ACC within a 7 to 14 days interval. This analysis will provide evidence of the feasibility, reliability and construct validity of the revised version of the QOL-ACC to assess QoL of older Australians accessing subacute care services.

### Stage 3 – development of a preference-based scoring algorithm

For the revised version of the QOL-ACC to be suitable for use in economic evaluation, it is necessary to develop a corresponding preference-based scoring algorithm, that enables the conversion of questionnaire responses into preference-based utility values. These utility values enable the calculation of quality-adjusted life years (QALYs) a key outcome for economic evaluation. The preference-based scoring algorithm will be generated through a Discrete Choice Experiment (DCE). DCEs are widely used in health economics to quantify the relative importance of different health and quality-of-life attributes or dimensions [9]. DCEs with survival duration (DCE_TTO_) have been shown to be a robust and flexible approach for valuing preference-based QoL measures and are increasingly adopted for the development of preference-based scoring algorithms for preference-based QoL measures [9, 34, 35]. The DCE_TTO_ method anchors relative preferences to the utility scale required for the calculation of QALYs by incorporation of a survival/duration attribute for each health/QoL state being valued [36]. The survey will be designed to accommodate two modes of administration: online administration and interviewer facilitation.

#### Participants and setting

Older adults (*N* = 1000) currently receiving, and/or with recent exposure to subacute care services (within the preceding 6 months) from all Australian states and territories will be recruited from the health system and via an online panel company. Participants will be aged 65 years and over and be able to read and understand English. Participants will be purposively sampled to maximise inclusivity to reflect a range of socio-demographic characteristics, and relevant experience with subacute care services, enabling them to provide meaningful judgements about different QoL states.

#### Recruitment

The recruitment process will be the same as the process outlined in stage one for the interview-facilitated version. This will be supplemented with recruitment through an online panel for the online survey. A target sample size of *N* = 1000 is considered sufficient to meet the requirements of a DCE_TTO_ experimental design. This sample size ensures accurate estimation of model parameters for development of the preference-based scoring algorithm for the revised QOL-ACC whilst also allowing for any extremes of heterogeneity in preferences [37].

#### Procedure

Participants will complete a survey that comprises of three main sections. The first section will involve completing the revised QOL-ACC to enable the participants to become familiar with the wording, and levels for the dimensions of the revised QOL-ACC. The second section will involve participants completing a DCE. Participants will be presented with a series of hypothetical response choice tasks on varying levels of the QOL-ACC domains. For each choice set, the participant will be asked to make a choice between two hypothetical QoL states, including survival duration (1, 4, 7 and 10 years). The third section will consist of a series of socio-demographic questions.

#### Analysis

Data will be analysed and modelled using a conditional logit model to estimate parameters interacting the dimension levels with continuous survival duration [35].

On completion of all three stages of the research, a validated preference-based QoL measure will be produced and will be available to be used in subacute care settings with older people to assess QoL.

## Discussion

Quality improvements are urgently needed across the health care system, particularly for older people who often have complex health needs, yet older people rarely have a voice. Despite being high users of subacute care services, no tailored measures for routine-system-wide monitoring and reporting of older people’s QoL exist. This important research study will address this gap by working with key health partners and older people to develop, validate and implement a new robust health economic measurement tool to assess QoL. This study will develop the first preference-based QoL measure tailored for older people receiving care in subacute care settings. Once developed, the measure will be readily available for use by researchers, clinicians, service providers and policymakers in Australia and internationally. By enabling the application of the QOL-ACC across both health and aged care systems, this informed decision-making can ensure that scarce resources are invested in models of care and service innovations that improve system efficiency while maximising QoL outcomes for older people.

### Dissemination

Upon completion of the research, the strengths and limitations of the study will be reported. Dissemination of the findings will be through project reports and peer-reviewed journal articles. Participants will have the option of receiving a one-page summary of the results. Findings will also be disseminated at relevant conferences.

## Data Availability

No datasets were generated or analysed during the current study.

## Abbreviations

QoL: Quality of life
QOL-ACC: Quality of Life – Aged Care Consumers
DCE: Discrete Choice Experiment
